# Graphene Oxide Loaded on TiO_2_-Nanotube-Modified Ti Regulates the Behavior of Human Gingival Fibroblasts

**DOI:** 10.3390/ijms23158723

**Published:** 2022-08-05

**Authors:** Xu Cao, Keyi Wu, Caiyun Wang, Yatong Guo, Ran Lu, Xin Wang, Su Chen

**Affiliations:** Laboratory of Biomaterials and Biomechanics, Beijing Key Laboratory of Tooth Regeneration and Function Reconstruction, Beijing Stomatological Hospital, Capital Medical University, Beijing 100050, China

**Keywords:** soft tissue integration, human gingival fibroblasts, titanium dioxide nanotubes, graphene oxide, MAPK pathway

## Abstract

Surface topography, protein adsorption, and the loading of coating materials can affect soft tissue sealing. Graphene oxide (GO) is a promising candidate for improving material surface functionalization to facilitate soft tissue integration between cells and biomaterials. In this study, TiO_2_ nanotubes (TNTs) were prepared by the anodization of Ti, and TNT-graphene oxide composites (TNT-GO) were prepared by subsequent electroplating. The aim of this study was to investigate the effect of TNTs and TNT-GO surface modifications on the behavior of human gingival fibroblasts (HGFs). Commercially pure Ti and TNTs were used as the control group, and the TNT-GO surface was used as the experimental group. Scanning electron microscopy, X-ray photoelectron spectroscopy, and X-ray diffraction were used to perform sample characterization. Cell adhesion, cell proliferation, cell immunofluorescence staining, a wound-healing assay, real-time reverse-transcriptase polymerase chain reaction (RT-PCR), and Western blotting showed that the proliferation, adhesion, migration, and adhesion-related relative gene expression of HGFs on TNT-GO were significantly enhanced compared to the control groups, which may be mediated by the activation of integrin β1 and the MAPK-Erk1/2 pathway. Our findings suggest that the biological reactivity of HGFs can be enhanced by the TNT-GO surface, thereby improving the soft tissue sealing ability.

## 1. Introduction

Titanium is widely used in the field of oral implantation due to its excellent mechanical properties and good biocompatibility [1,2,3]. However, some implants still fail because of poor osseointegration, bacterial infection, and poor adhesion to soft tissue [4,5,6]. Most of the current research focuses on the osteogenesis and antibacterial aspects of implants, while less attention has been paid to soft tissue sealing [7,8,9]. In fact, the long-term stability of implants is related to both osseointegration and the sealing effect of soft tissue. An ideal soft tissue seal forms an epithelial cuff and connective tissue loop [10], and the collagen fibers between the implant and the soft tissue are tightly and firmly connected. This is crucial in implant restoration because a good soft tissue seal can form an effective protective barrier, inhibit bacterial invasion and the apical migration of the junctional epithelium, and reduce the risk of bone loss [11,12,13,14]. Hence, it is of great importance to develop methods to improve the soft tissue sealing capabilities of materials.

The proliferation and initial adhesion of human gingival fibroblasts (HGFs), the main cells in the soft tissue of the transmucosal area, are critical for peri-implant mucosal healing [15]. HGFs can secrete the main components of the extracellular matrix (ECM), such as collagen and fibronectin, to promote soft tissue regeneration and attachment, facilitate the formation and remodeling of the ECM, and play an important role in inflammatory and bacterial immune responses [16,17]. Therefore, HGFs are key cells that determine the bond between the connective tissue and implant and are important for peri-implant soft tissue sealing.

Previous studies have shown that the surface features of the transgingival area of the implant, such as the surface morphology and physical and chemical properties, can affect the adhesion and growth of fibroblasts [18,19,20]. As a result of the nanotopography, HGFs can adhere better, and gingival connective tissue can be inserted more easily into the nanostructures to maintain a similar orientation as the collagen fibers in the periodontal tissue [21,22,23]. In addition, the surface hydrophilicity and protein adsorption ability are key factors that control the response of cells to biomaterials. HGFs tend to attach and proliferate on hydrophilic surfaces, which are beneficial for enhancing the attachment of connective tissue to the abutment [24,25,26]. An increase in protein adsorption may also promote the early adhesion of fibroblasts [27]. Therefore, the hydrophilicity and protein adsorption ability of the material can be promoted by modifying the surface of the material, thereby promoting the adhesion and proliferation of fibroblasts.

Graphene oxide (GO) [28,29] is a derivative of graphene with abundant functional groups. These features impart GO with good hydrophilicity, dispersibility, and biocompatibility. The π–π bond stacking of GO can adsorb nutrients and promote cell adhesion, proliferation, and migration [30], where highly wrinkled cross-linked GO has been shown to regulate cytoskeletal tension to promote cell adhesion [31]. These advantages mean that GO has broad application prospects in the biomedical field. However, few studies have focused on using GO to enhance the soft tissue sealing capabilities of dental implants. Therefore, the effect of GO on HGFs needs further study.

The modification of TiO_2_ nanotubes (TNTs) by GO may further promote the hydrophilicity and protein adsorption of the material and promote the adhesion, proliferation, and migration of HGFs, thereby promoting soft tissue sealing and the long-term stability of the implant. In this study, GO was loaded onto TNT-modified Ti sheet samples via electroplating, and the possible mechanism of GO in soft tissue sealing was investigated.

## 2. Results

### 2.1. Surface Properties

**Figure 1:**
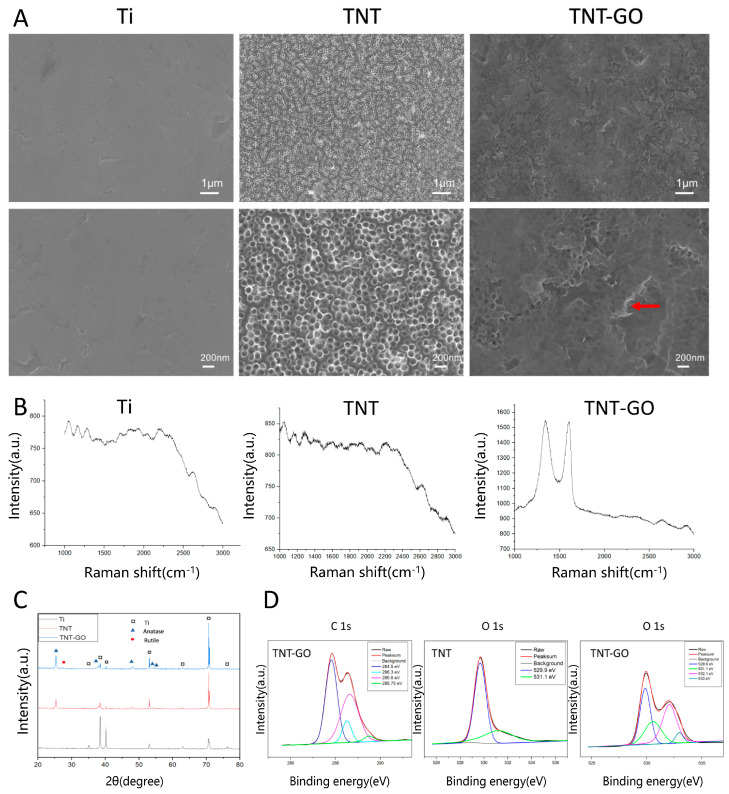
Results of surface characterization of the Ti, TNT, and TNT-GO samples. (**A**) SEM images; (**B**) Raman spectra; (**C**) XRD patterns; (**D**) High—resolution XPS spectra of C 1s and O 1s. Abbreviations: Ti, pure titanium; TNT, TiO_2_ nanotubes; TNT-GO, TiO_2_ nanotubes with graphene oxide; XRD, X-ray diffraction; XPS, X-ray photoelectron spectroscopy. GO film was marked by red arrow.

Pure titanium sheets (Cuibolin Nonferrous Metal Industry Co., Ltd., Beijing, China) with dimensions of 1 cm × 1 cm were used as a blank control group, TNTs were used as a control group, and TNTs loaded with GO (TNT-GO) were used as the experimental group. The results of the scanning electron microscopy (SEM) are shown in Figure 1A. The surface of the Ti sample was relatively smooth, and a few scratches were observed. The surface of the TNT sample had a uniform TNT array with a diameter of approximately 100 nm. The SEM images of the TNT-GO sample showed that GO covered the surface of the nanotubes as a thin film (red arrow). The Raman spectra of the samples are shown in Figure 1B. The Raman spectrum of the TNT-GO specimen had peaks characteristic of GO, proving that GO was successfully loaded on the nanotubes. The D peak, which is related to the disordered vibration of graphene, was observed at approximately 1350 cm^−1^, and the G peak, which is the main characteristic peak of graphene, appeared around 1580 cm^−1^. X-ray diffraction (XRD) was performed on each group of samples to determine their crystal structures. Figure 1C shows that only the diffraction peaks of the Ti substrate were observed for the Ti group, as expected. After using the anodization method and heat treatment at 550 °C to prepare the TNT array, the diffraction peaks of the Ti substrate were still observed but with different peak intensity ratios compared to the blank Ti. The strong anatase TiO_2_ diffraction peaks indicate that the TiO_2_ nanotubes were transformed from amorphous to crystalline anatase TiO_2_ after the heat treatment. The crystal form of the TNTs did not change after GO deposition.

**Figure 2:**
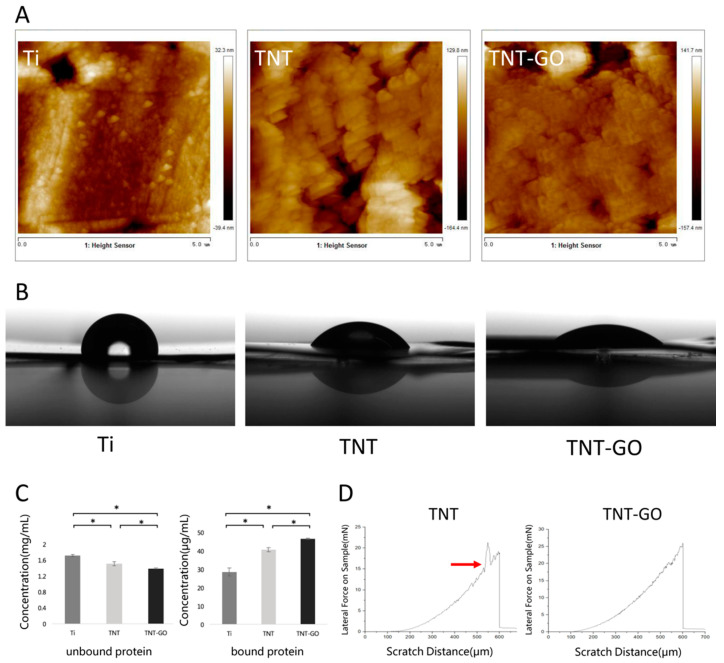
Surface analysis of the Ti, TNT, and TNT-GO samples. (**A**) Reconstructed topographical images of the samples determined from atomic force microscopy; (**B**) water contact angles; (**C**) unbound and bound protein concentrations; (**D**) nanoscratch results for TNT and TNT-GO. Red arrow and force fluctuations indicate fracture at a displacement of 530 μm. (* *p* < 0.05).

The high-resolution C1s peaks of the X-ray photoelectron spectroscopy (XPS) in the TNT-GO group were deconvoluted into four peaks at approximately 284.5, 286.3, 286.6, and 288.75 eV (Figure 1D), which are ascribed to C–C/C–H, C–OH, C=O/C-O, and O=C–O species, respectively [32]. The high-resolution O 1s peaks of the TNT group were resolved into two peaks at 529.9 and 531.1 eV (Figure 1D), which are attributed to Ti-O and Ti-OH species, respectively [33]. The high-resolution O 1s peaks of the TNT-GO group were resolved into four peaks at approximately 529.9, 531.1, 532.1, and 533 eV, which are attributed to Ti-O, Ti-OH/C=O, C-O, and C-OH, respectively [32,34], further proving the loading of GO. The surface roughness (Ra) of each group of specimens was detected using atomic force microscopy (AFM). Figure 2A and Table 1 show that the roughness of Ti was 9.5 ± 1.6 nm. The roughness values of TNT (46.5 ± 2.8 nm) and TNT-GO (42.1 ± 3.3 nm) were similar. The results for surface hydrophilicity are shown in Figure 2B and Table 1. The surface water contact angle of Ti was 90.9 ± 3.4°, while that of TNT was 46.3 ± 2.8° and that of TNT-GO was 33.9 ± 6.3°; there was a statistical difference among the groups. The results for the unbound and bound proteins are shown in Figure 2C. The amount of protein adsorption increased in the order of Ti, TNT, and TNT-GO. The nanoscratch test results are shown in Figure 2D. The transition point of the scratch curve of each group from smooth to large fluctuations indicates the point of coating peeling (interface fracture), and the load measured at this point is the critical load for failure. The TNT group showed obvious fluctuations and fractures at a displacement of 530 μm (Figure 2D, red arrow), and its critical load was approximately 98.75 mN. However, no obvious exfoliation or fracture were observed for the TNT-GO group.

### 2.2. Cell Adhesion and Proliferation

**Figure 3:**
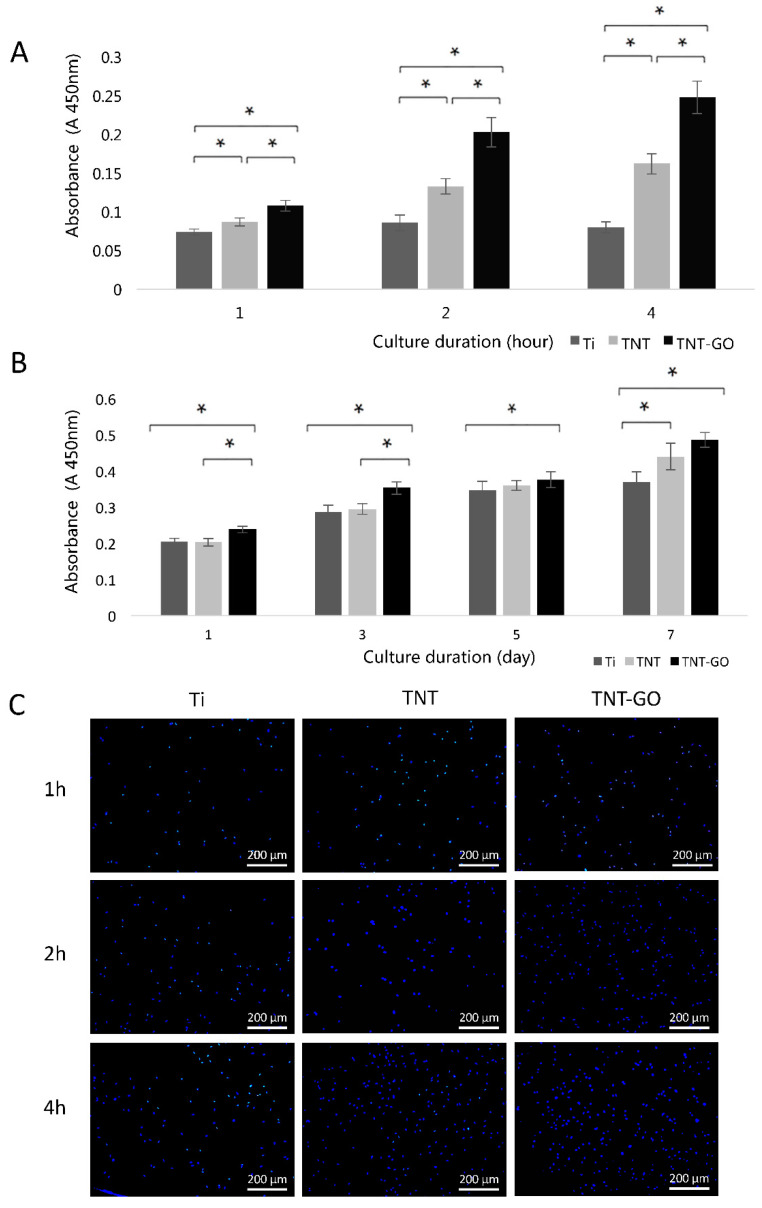
(**A**) Cell adhesion of human gingival fibroblasts (HGFs) on the samples after 1, 2, and 4 h. (**B**) Cell proliferation of HGFs after 1, 3, 5, and 7 d. (**C**) DAPI staining of adherent HGFs after 1, 2, and 4 h. (* *p* < 0.05).

The adhesion of the HGFs to the different specimens is shown in Figure 3A. In the case of Ti, the number of adhered cells did not change significantly over time. The number of cells adhered to the surface of TNT-GO increased over time, and at each time point, the number of adherent cells in the TNT-GO group was higher than those in the other two groups. The proliferation ability of the HGFs is shown in Figure 3B, where the proliferation activity in each group increased over time. On days 1, 3, 5, and 7, the proliferation of HGFs on the surface of TNT-GO was higher than that of Ti (*p* < 0.05), indicating that the loading of GO could promote the proliferation potential of HGFs. Figure 3C shows that the HGFs in the TNT-GO group adhered better than those in the other two groups, which is consistent with the previous trend of the cell-counting Kit-8 assay (CCK-8) results.

### 2.3. Cell Morphology

**Figure 4:**
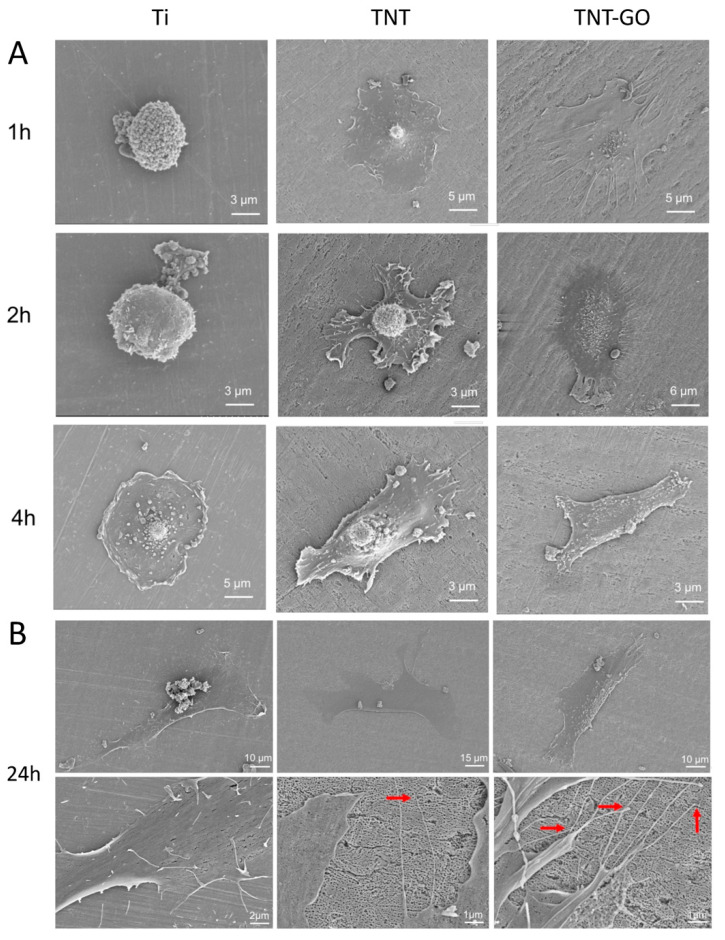
Scanning electron microscopy (SEM) images of HGFs on the different surfaces. (**A**) 1, 2, and 4 h. (**B**) 24 h. Red arrows indicate filopodia anchorage within nanotubes.

The morphology and adhesion of the cells were observed at 1, 2, 4, and 24 h by SEM (Figure 4). The cells on the surface of Ti were round at 1 and 2 h and did not spread. HGFs seeded on the TNT group spread in a disc shape and began to protrude pseudopodia. HGFs seeded on the TNT-GO group spread in a polygonal shape with obvious cellular pseudopodia, and the pseudopodia were more numerous, longer, and tightly attached compared to those on the TNT array. At 4 h, the cells in the Ti group spread in a disc shape, while those in the TNT and TNT-GO groups spread in a spindle shape with good adhesion. At 24 h, the HGFs on the surfaces of the three groups exhibited fusiform or polygonal spreading (Figure 4B). Under high magnification, cell filopodia were observed clearly for the TNT group, and the edges were attached to the nanotubes. In comparison, the HGFs in the TNT-GO group had more filopodia tightly anchored in the nanotubes, with some extending deep into the nanotubes (indicated by the red arrows).

### 2.4. Immunocytochemistry

**Figure 5:**
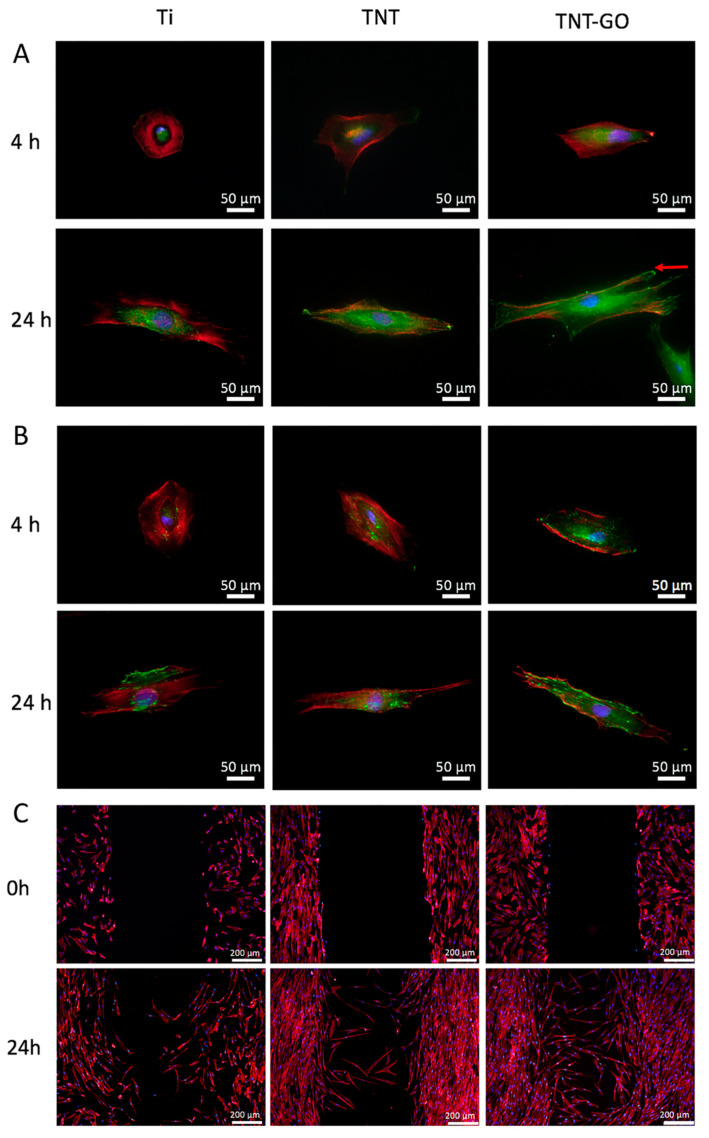
Immunofluorescence staining of HGFs on the different surfaces at 4 h and 24 h. F-actin was labeled red, and nuclei were labeled blue. (**A**) Vinculin (green); (**B**) integrin β1 (green). (**C**) Wound-healing assays at 0 and 24 h. Red arrow indicates punctate mature focal adhesion sites expressed at the edge of the pseudopodia.

The immunofluorescence staining results at 4 and 24 h are shown in Figure 5A,B. The results of vinculin (VCL) staining are shown in Figure 5A. Red fluorescence indicates the F-actin-labeled cytoskeleton, green fluorescence indicates the focal adhesion protein (VCL), and blue fluorescence indicates the nucleus. At 4 h, the HGFs in the Ti group were nearly round, and the green fluorescence of VCL was only slightly expressed and concentrated in the nucleus. In the TNT and TNT-GO groups, HGFs spread in an elongated spindle shape, and the VCL protein was observed in greater abundance. The distribution of the VCL protein in the TNT-GO group extended with the extension of the cell shape, with a wider distribution than on TNTs. After 24 h, the cells were spread on the surfaces of the three groups. In the TNT group, the VCL protein was also significantly expressed at the cell edge, which was more obvious for the TNT-GO group. The punctate and long mature VCL proteins were expressed over a large area at the edge of the pseudopodia (indicated by the red arrow), and the green fluorescent region occupied most of the main body of the cells for the TNT-GO group.

The immunofluorescence staining results for the integrin beta 1 antibody (ITGB1) are shown in Figure 5B. The green fluorescence corresponds to ITGB1. At 4 h, ITGB1 was only formed in small amounts and was distributed close to the nuclei. The expression of ITGB1 was significantly enhanced in TNT-GO compared to the other samples and extended from the nucleus to the edge of the cell. At 24 h, the expression of ITGB1 in the TNT-GO group was significantly higher than the Ti and TNT groups, and the edges of the pseudopodia were also clearly expressed in punctate forms (indicated by the red arrows), with green fluorescence occupying most of the main body of the cell.

### 2.5. Wound-Healing Assay

The effect of the material surface on the migration of HGFs was studied using scratch experiments and phalloidin fluorescent staining. Figure 5C shows that HGFs cultured for 24 h after scratching the surface of the Ti group had a slow migration speed and low mobility, whereas the HGFs on the surface of the TNT group had a faster migration speed than on pure Ti. The TNT-GO group had the strongest cell migration ability, and a large number of cells migrated to the center of the scratch.

### 2.6. Gene Expression

**Figure 6:**
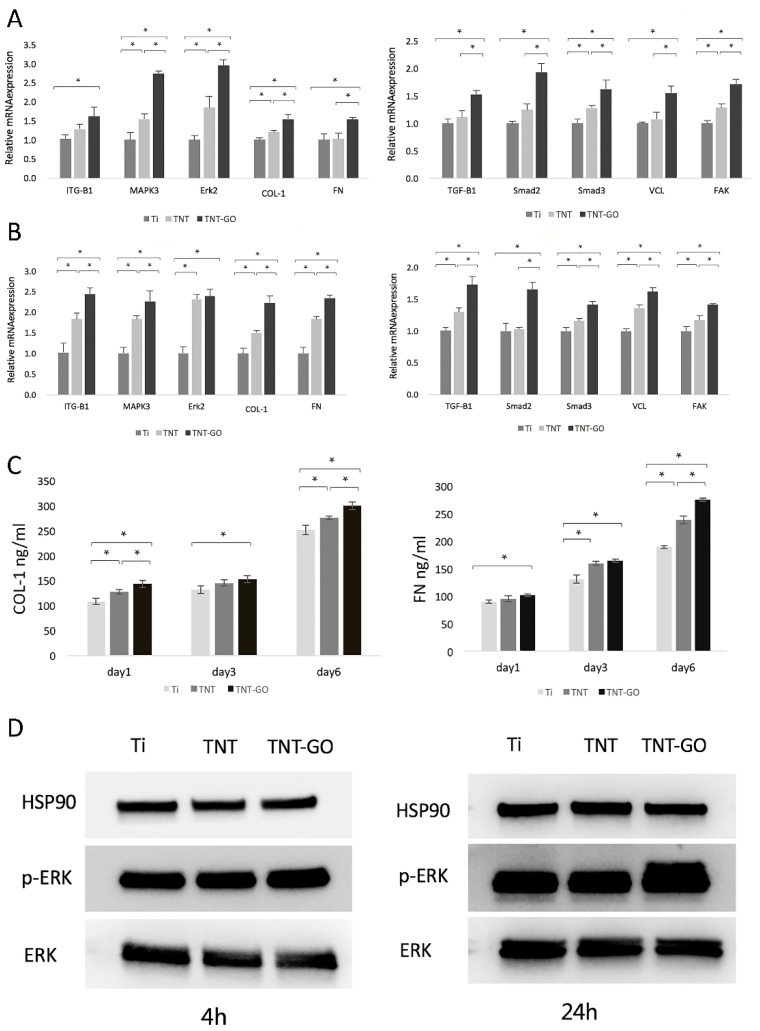
Gene expression of HGFs on the different surfaces after (**A**) 4 h and (**B**) 24 h; (**C**) ELISA results of COL-1 and FN in HGFs at 1, 3, and 6 d; (**D**) Western blot of ERK and p-ERK at 4 h and 24 h (* *p* < 0.05).

The results of the relative gene expression of the HGFs in the three groups are shown in Figure 6. At 4 h (Figure 6A), there were significant differences in *FAK, COL-1, MAPK3, Erk2*, and *Smad3* expression among the three groups. The mRNA expression levels of *FN*, *TGFB1, VCL*, and *Smad2* in the TNT-GO group were higher than the Ti and TNT groups (*p* < 0.05), and *ITGB1* was also significantly increased.

At 24 h (Figure 6B), the relative gene expressions of *VCL, FAK, FN, COL-1, ITGB1, MAPK3, TGF-B1,* and *Smad3* in HGFs cells on the surfaces of the three groups were all significantly different. In addition to *Erk2, TGF-B1, Smad2,* and *Smad3*, which are related to cell proliferation, also significantly increased in the TNT-GO group.

### 2.7. Enzyme-Linked Immunosorbent Assay (ELISA)

The ability of the HGFs in each group to bind collagen type 1 (COL-1) and fibronectin (FN) was detected using ELISA (Figure 6C). The expression levels of FN and COL-1 in the TNT-GO group were higher compared to the Ti group at every time point, with statistical differences. On days 1 and 6, COL-1 secretion varied significantly among the three groups. On day 6, FN secretion varied significantly among the three groups.

### 2.8. Western Blot

The expression levels of Erk and p-Erk in the HGFs are shown in Figure 6D. The results showed that at 4 and 24 h the expression of p-Erk in the TNT-GO group was significantly upregulated, indicating that the TNT-GO group was more likely to activate the phosphorylation of Erk in HGFs than the other surfaces.

### 2.9. Response of HGFs after Blocking Erk1/2 Signaling

**Figure 7:**
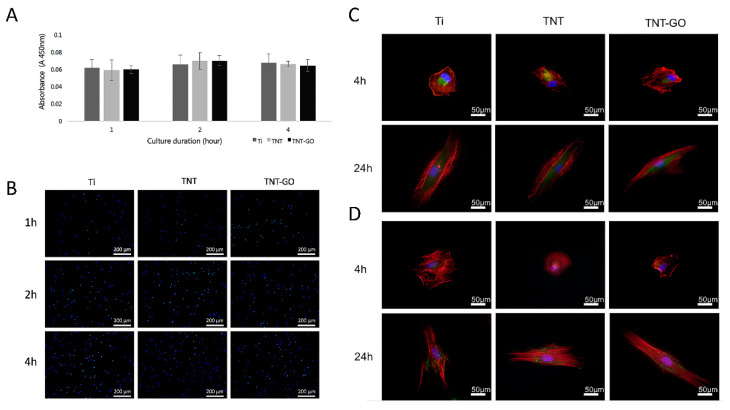
Cell analysis at 1, 2, and 4 h after inhibiting the Erk1/2 signaling pathway. (**A**) Cell adhesion of HGFs; (**B**) DAPI staining of adherent HGFs; immunofluorescence staining of (**C**) vinculin and (**D**) integrin β1.

After using 20 μM PD98059 to inhibit the Erk1/2 signaling pathway, the adhesion and expression of related genes and proteins were detected. The adhesion of HGFs on the three groups of specimens showed no significant difference (Figure 7A,B). The immunofluorescence staining expression of VCL and integrin β1 (Figure 7C,D) was significantly reduced compared to that before inhibition (Figure 5A,B), and the three groups showed no significant differences.

**Figure 8:**
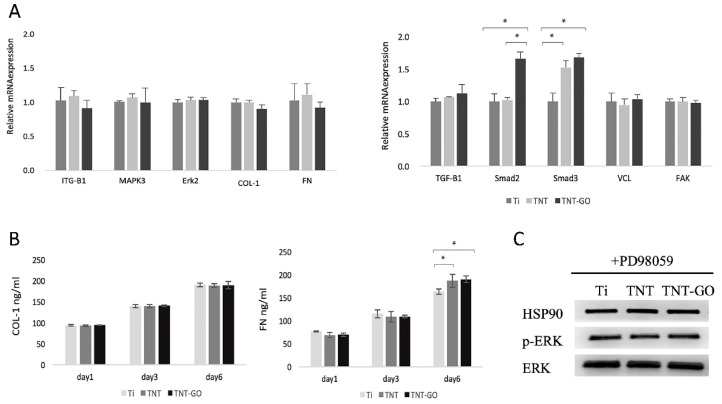
RT-PCR, ELISA, and Western blot results after inhibiting the Erk1/2 signaling pathway. (**A**) RT-PCR results of gene expression in HGFs at 24 h; (**B**) ELISA of COL-1 and FN in HGFs at 1, 3, and 6 d; (**C**) Western blot results of ERK and p-ERK at 24 h (* *p* < 0.05).

The expression of the adhesion-related genes (Figure 8A) *VCL, FAK, FN, COL-1, ITGB1, MAPK3, and ERK2* was not significantly different for the three groups at 24 h, while the expression of the cell proliferation-related genes *Smad2* and *Smad3* was higher in the TNT-GO group. The ELISA results (Figure 8B) showed that there was no significant difference in COL-1 secretion at 1, 3, and 6 d after inhibiting the Erk1/2 signaling pathway, while the secretion of FN was slightly higher in the TNT and TNT-GO groups at day 6. The phosphorylation of ERK 1/2 of HGFs on the surface of the three groups was significantly inhibited after inhibiting the Erk1/2 signaling pathway (Figure 8C).

## 3. Discussion

Effective surface modification and implant abutment design can favor soft tissue response [18,35,36], resulting in strong connective-tissue attachment, which is beneficial for long-term implant stability. In this study, we performed in vitro studies to investigate the effect of surface modification on HGFs.

Surface morphology, roughness, hydrophilicity, and the protein adsorption of implant materials can influence the biological behavior of cells, including their adhesion, spreading, migration, and proliferation on the surface [25,27]. Several studies have shown that nanotopography can promote cell adhesion and proliferation [37,38]. This was also confirmed by our SEM (Figure 4) and fluorescence staining results (Figure 5A,B), which showed that TNTs promoted the adhesion and spreading of HGFs and cytoskeleton elongation. This may be due to the nanotopography providing cells with a biomimetic environment, allowing HGFs to form more focal adhesion (FA) attachment sites at the nanotube edges [39,40]. In addition, increased roughness promoted cell adhesion, which is consistent with the results of previous studies [41,42,43,44]. The cell adhesion on the TNT-GO group was better than that of the TNT group, implying that, in addition to the nanotopography, the GO provided better protein adsorption and a biocompatible nanoscale [45]. Moreover, the roughness of the TNT and TNT-GO groups was much lower than the Ra threshold of 0.2 μm, above which bacterial adhesion is enhanced [46].

Studies have shown that after biomaterials are implanted into the body, they first undergo hydration with water molecules very quickly, and then small molecular proteins first adhere to the surface of the implant, followed by the exchange and adsorption of protein molecules, i.e., the Vroman effect [47]. Protein molecules with high affinity replace the initially adhered small protein molecules, form a tightly bound protein layer, guide the adhesion of cells to the surface of the material, and determine the biological properties of the surface of the material [48,49]. Therefore, the protein adsorption behavior on the material surface determines the properties of the implant–tissue interface. Compared to the Ti group, the protein adsorption of the TNT group was significantly improved. After loading with GO, the protein adsorption of the material increased further. This may be due to the enhanced adsorption provided by GO itself, in addition to the hydrophilic surface formed after loading GO. Previous studies have shown that hydrophilic surfaces can adsorb more BSA than hydrophobic ones [50,51]. Other studies have shown that hydrophilic surfaces are more favorable for protein adsorption and cell adhesion, and such surfaces favor the replacement of pre-adsorbed albumin by cell adhesion proteins [52].

Using SEM to observe the cell morphology and spreading of HGFs, it can be seen that after 1–24 h of culture, the HGFs in the TNT and TNT-GO groups showed better cell adhesion and spreading compared with the Ti group, especially for the TNT-GO group with pronounced cell pseudopodia and many filopodia extending outward and into the nanotubes. Differences in the protein adsorption capacity and hydrophilicity of the three samples may be responsible for the differences in the adhesion and spreading of HGFs [27]. Compared with the other two groups, the TNT-GO group had the highest hydrophilicity and protein adsorption (Figure 2B,C), and because GO itself can adsorb nutrients in the medium, it may further promote cell adhesion.

Interactions between cells and ECM proteins on the surfaces of biomaterials can affect cell proliferation, adhesion, and cell morphological changes [53]. The COL-1 in the ECM can regulate cell migration, adhesion, and gene expression in response to HGFs [54]. As a ligand of the integrin receptor family, FN affects the distribution of the cytoskeleton [53,55]. COL-1 and FN can establish a firm soft tissue seal between the implant and the tissue, modulate the ECM composition, and influence HGF adhesion, morphology, and migration by building collagen networks [56]. The ELISA results showed that the secretion of Col-I and FN in the TNT-GO group was significantly higher than that in the Ti (Figure 6C).

The TNT-GO group could modulate the behavior of HGF by promoting the secretion and accumulation of Col-I and FN, where HGFs in the TNT-GO group showed better adhesion, migration, and proliferation compared to the other surfaces. To better study the mechanism by which the TNT-GO group affected the behavior of HGFs, we performed Western blot and RT-PCR assays. The PCR results showed that the *ITGB1, MAPK3*, and *Erk2* levels in the TNT-GO group were higher than those in the Ti group (Figure 6A,B). Integrin receptors regulate cell adhesion and migration by connecting the internal structures of cells to external environments [27,57]. The expression level of *ITGB1* in HGFs was higher on the TNT-GO group than in the Ti and TNT group. This may be related to the π-π bond stacking and surface morphology of GO, which can adsorb more nutrients and promote cell adhesion [30]. Combined with the ITGB1 fluorescent staining results (Figure 5B), this suggests that by stimulating ITGB1 expression GO can influence the movement and adhesion of HGFs.

ITGB1 was reported to mediate cell adhesion [58,59], and graphene promoted the protein expression of ITGB1 in MSCs on graphene-coated substrates [60]. Graphene with complex wrinkled and corrugated morphologies mimics the morphology of disordered nanopit arrays [61]. This is thought to promote protein adsorption, cell adhesion, proliferation, and differentiation [60], and the porous folded topography of the surface of graphene or GO can mechanically stimulate cells and initiate cascade reactions [62]. Moreover, integrin α5/β1 has been shown to activate ERK1/2, p38, and JNK MAPK during the osteogenic differentiation of embryonic stem cells (ESCs) in response to polydopamine (PDA) /GO [63]. Previous studies have shown that ITGB1 [64] regulates cell motility and adhesion capacity by activating the MAPK signaling pathway [27], which was validated by the enhanced gene expression of MAPK and Erk2 in the TNT-GO group in this study. The phosphorylation level of Erk1/2 in the TNT-GO group was significantly increased compared to the other surfaces, which proved that GO activated the MAPK signaling pathway and promoted the phosphorylation of Erk1/2 to regulate the movement and adhesion of HGFs. In addition, the proliferation of HGFs promoted by TNT-GO may be related to the increased expression of *TGFB1, Smad2, and Smad3* (Figure 6B). Therefore, the TNT-GO group may stimulate a massive accumulation of ECM due to the high expression level of integrins and the activation of the MAPK-Erk pathway, creating a microenvironment on the sample surface that is favorable for cell adhesion and migration.

Through an in vitro experimental analysis, we found that, compared with the Ti and TNT groups, TNT-GO could modulate the behavior of HGFs to promote cell adhesion, proliferation, and migration, which may further promote soft tissue sealing. In addition, osseointegration and a reduction in bacterial invasion can also affect implant stability. In previous literature, GO has been shown to have antibacterial properties and promote osseointegration [65,66], and the nanotube structure also contributes to osseointegration [38]. Therefore, TNT-GO may have positive effects on osseointegration and antibacterial properties, which need to be further explored.

## 4. Materials and Methods

### 4.1. Sample Preparation

The pure Ti sheets were ultrasonically cleaned sequentially using acetone, ethanol, and deionized water for 10 min each. TNTs were prepared on the Ti sheet surface using the anodization method. Briefly, a pure Ti sheet was anodized (50 V, 15 min) in ethylene glycol electrolyte (containing 10 vol% deionized water and 0.5 wt% ammonium fluoride) and annealed at 550 °C for 2 h to obtain an array of TNTs on the Ti surface. Then, single-layer flake GO (0.1 mg/mL GO, 100681, XFNANO, Nanjing, China) was ultrasonically dispersed for 2 h before use and then loaded on the surface of the TNTs by electroplating at a loading voltage of 50 V for 5 min to prepare TNT-GO.

### 4.2. Sample Characterization

The surface topography of the materials was observed by SEM (S4800, Hitachi, Ltd., Tokyo, Japan). A Raman spectrometer (LabRAM HR800, HORIBA, Paris, France) was used at the 532 nm line as the excitation source to determine the sample structure in the 1000–3000 cm^−1^ range. XRD (TTRAX III, Rigaku Co., Tokyo, Japan) was used to analyze the crystal phases. XPS (ESCALAB 250Xi, Thermo Fisher Scientific, MA, USA) was used to detect the chemical states of the samples. A nanoscratch experiment was used to determine the bonding strength of the materials (Keysight G200, Keysight Technologies Co., Ltd., Beijing, China). The surface roughness of the samples was analyzed using AFM (Nanoscope V, Veeco, Santa Barbara, CA, USA). The contact angles of the samples were measured using an optical contact-angle measurement device (OCA15pro, Filderstadt, Germany).

### 4.3. Protein Adsorption

The protein adsorption of Ti, TNTs, and TNT-GO was detected using bovine serum albumin (BSA, Sigma-Aldrich Co., St. Louis, MO, USA) as a model protein. Each group of samples was placed in a 24-well plate, and 200 μL of protein solution (2 mg/mL BSA) was added. After incubation for 1 h, 20 μL of supernatant was collected from each well to evaluate the amount of unbound protein. The samples were washed thrice with phosphate-buffered saline (PBS, Biosharp, Hefei, China). Then, 200 μL of 2% sodium dodecyl sulfate (SDS, Beyotime, Shanghai, China) was added to the sample surface to elute proteins under shaking for 2 h. A BCA protein detection kit was used to detect the protein concentration. A microplate spectrophotometer was used to determine the optical density (OD) at 562 nm.

### 4.4. Cell Culture

Commercial HGFs (CRL-2014, ATCC) were purchased from ATCC (Manassas, VA, USA) and were cultured (37 °C, 5% CO_2_) in Minimum Essential Medium α containing 10 vol% fetal bovine serum (FBS) and 1 vol% penicillin/streptomycin; all these reagents were supplied by Thermo Fisher Scientific, Waltham, MA, USA. Passages 3–6 of HGFs were used in this experiment.

### 4.5. Cell Proliferation and Adhesion

The cell-counting Kit-8 assay (CCK-8, Dojindo, Kyushu Island, Japan) was used to detect the adhesion and proliferation of HGFs. HGFs were seeded on the samples to detect proliferation after 1, 3, 5, and 7 d and were then incubated with CCK8 for 2 h. A microplate spectrophotometer was used to determine the OD at 450 nm. 

Next, 3 mL of cell suspension was added along with the sample into a centrifuge tube to determine the adhesion ability of HGFs [25]. The centrifuge tubes were fixed in an incubator and rotated constantly. CCK-8 was used to determine the number of adherent cells after 1, 2, and 4 h of culture. 

Fluorescence microscopy (OLYMPUS, Tokyo, Japan) was used to observe 4′,6-diamidino-2-phenylindole (DAPI, ZLI-9557, ZSGB-BIO, Beijing, China)-stained early-adhered nuclei (magnification ×10).

### 4.6. Cell Morphology

The cell spreading morphology was detected using SEM (Hitachi S-3400, Tokyo, Japan). The HGFs on each sample were incubated for 1, 2, 4, and 24 h and fixed with 2.5% glutaraldehyde overnight. All samples were then continuously dehydrated and sputter-coated with platinum for the next observation.

### 4.7. Immunofluorescence

To evaluate the expression of HGF cell-adhesion-related proteins on the surfaces of different materials, specific immunostaining with VCL and ITGB1 was performed. HGFs were seeded on samples in 24-well plates, then fixed with 4% paraformaldehyde after 4 and 24 h of incubation, permeabilized with 0.1% Triton X-100, blocked with goat serum blocking solution (ZLI-9056, ZSGB-BIO, Beijing, China) for 1 h, and incubated overnight with specific primary antibodies targeting VCL or ITGBI (Abcam, Cambridge, UK). After washing, the membranes were incubated with the secondary antibody for 1 h in the dark. Actin staining was performed after incubation with Alexa Fluor 555 phalloidin (Cell Signaling Technology, Boston, MA, USA) for 1 h. Nuclear staining was performed using DAPI. Images were obtained using a confocal laser-scanning microscope (Leica, Hamburg, Germany).

### 4.8. Wound-Healing Assay

The HGFs were seeded onto the specimens. After reaching confluency, the cell monolayer was carefully damaged using a plastic pipette and cultured in FBS-free medium for 24 h. Then, the specimens were fixed in 4% paraformaldehyde and permeabilized with 0.1% Triton X-100. Actin staining was performed using Alexa Fluor 555 phalloidin, and the nuclei were stained with DAPI. Confocal laser scanning microscopy was used to observe migration.

### 4.9. Western Blot Tests

Radioimmunoprecipitation assay lysis buffer (Beyotime, Shanghai, China) was used to lyse cells and extract total protein. Then, 25 μg of protein was loaded on a 10% sodium dodecyl sulfate polyacrylamide gel and then transferred to polyvinylidene fluoride membranes using a Trans-Blot Turbo system (Bio-Rad, Hercules, CA, USA). Next, 5% dehydrated milk was used to block the membranes, then the primary anti-phosphorylated Erk1/2 (Abclonal, Wuhan, China) and anti-Erk1/2 (Abclonal, Wuhan, China) antibodies were incubated with the membranes overnight. The membranes were then incubated with horseradish peroxidase goat anti-rabbit IgG (Abclonal, Wuhan, China) and visualized with Clarity™ Western ECL Substrate (Bio-Rad, Hercules, CA, USA). The primary monoclonal antibody used as the housekeeping protein was a monoclonal antibody against heat shock protein 90 (HSP90, Abclonal, Wuhan, China).

### 4.10. ELISA

The ability of the HGFs to secrete COL-1 and FN proteins was determined using an ELISA kit (Cusabio Technology, Wuhan, China). HGFs were seeded on the samples and incubated for 1, 3, and 6 d. The cell culture supernatants of each sample were collected, and an ELISA assay was performed. The concentrations of COL-1 and FN were quantified at 450 nm.

### 4.11. Quantitative RT-PCR Analysis

Adhesion- and proliferation-related gene expression levels were analyzed by RT-PCR. HGFs were seeded onto specimens and incubated for 4 and 24 h. Trizol reagent (Invitrogen, Carlsbad, CA, USA) was used to extract total RNA, which was then reverse-transcribed using a Prime Script RT kit (TaKaRa, Tokyo, Japan). A number of gene expressions were detected, including *Erk2, ITGB1, MAPK3, FAK, TGF-β, VCL, Col-I, FN, Smad3,* and *Smad2*. The primer sequences used are listed in Table 2. The GAPDH expression levels were used to normalize all mRNA values. The relative gene expression was calculated using the 2^−∆∆Ct^ method [67].

### 4.12. Responses of HGFs after Blocking Erk1/2 Signaling

In order to investigate whether Erk1/2 was responsible for the enhanced adhesion of the TNT-GO group, we used a specific inhibitor, PD98059 (20 μM, Cell Signaling Technology, Boston, MA, USA), to block Erk1/2 signaling. The adhesion of HGFs and the expression of related genes and proteins was detected after Erk1/2 signaling was inhibited.

### 4.13. Statistical Analysis 

A one-way ANOVA (*p* < 0.05) was used to determine statistical significance for all data using SPSS software (version 26.0, IBM Corp., Chicago, IL, USA).

## 5. Conclusions

In this study, we used anodic oxidation to prepare TiO_2_ nanotubes and loaded GO by electroplating, and we found that nanotube arrays and GO can modulate the cellular behavior of HGF on titanium surfaces. The monolayer GO-modified TiO_2_ nanotubes can regulate the expression of ITGB1 and activate the MAPK signaling pathway to regulate the adhesion, spreading, and migration of HGFs, possibly by promoting the expression of TGF-β1 to promote the proliferation of HGFs. In addition, TNT-GO promoted the synthesis of extracellular matrix and upregulated the expression of adhesion- and proliferation-related genes. Therefore, the surface modification of Ti with TNT-GO may be a promising method to promote soft tissue sealing in dental implants, which may promote better and faster soft tissue healing. More in vivo experiments are needed for further verification.

## Figures and Tables

**Table 1:** Water contact angles and surface roughness of the samples.

Sample	Treatment	Contact Angle (°)	Roughness (nm)
1	Ti	90.9 ± 3.4	9.5 ± 1.6
2	TNT	46.3 ± 2.8	46.5 ± 2.8
3	TNT-GO	33.9 ± 6.3	42.1 ± 3.3

Note: Data are expressed as means ± standard deviations (SD).

**Table 2:** Primer pairs for RT-PCR.

Gene	Primers Sequence (F = Forward, R = Reverse)
*ITGB1*	F: GCATCCCTGAAAGTCCCAAG R: CAATGGAGAGTGCGTCTGCG
*MAPK3*	F: GGACCTGATGGAGACTGACCTG R: GTTGGCGGAGTGGATGTACTT
*Erk2*	F: CTCATCCTCGGAAAACAGACC R: AATGCTGTGTGGACCTTCAGA
*FN*	F: GTGAACGACACATTCCACAAG R: GGTGGAAGTGTGATCCCGTCG
*Col-I*	F: GGCTCCCTCCTAGTCTGTCCT R: GGGAGGAAGCAAAAGACTCT
*TGF-β*	F: ACCTGAACCCGTGTTGCTCT R: CGCCAGGAATTGTTGCTGTA
*Smad2*	F: AACAGATGGGATGCTTCAGGT R: CCTCACTTGGCTTGCTTCTTT
*Smad3*	F: GAAGGAGGGTTGACTCAGAAC R: TTTCACACCAGGCACATACTT
*VCL*	F: CGAATCCCAACCATAAGCAC R: CGCACAGTCTCCTTCACAGA
*FAK*	F: CTCCTACTGCCAACCTGGAC R: GCCGACTTCCTTCACCATAG
*GAPDH*	F: ATTTGGTCGTATTGGGCGCC R: ACCTCAACTACATGGTTTAC

## Data Availability

Not applicable.
